# A Spatio-Temporal Dataset for Satellite-Based Landslide Detection

**DOI:** 10.1038/s41597-025-06167-2

**Published:** 2025-11-11

**Authors:** Paul Höhn, Konrad Heidler, Robert Behling, Xiao Xiang Zhu

**Affiliations:** 1 Data Science in Earth Observation, Technical University of Munich (TUM), 80333 Munich, Germany; 2 Remote Sensing Technology Institute, German Aerospace Center (DLR), 82234 Weßling, Germany; 3 GFZ Helmholtz Centre for Geosciences, 14473 Potsdam, Germany; 4 Munich Center for Machine Learning (MCML), 80333 Munich, Germany

**Keywords:** Natural hazards, Planetary science

## Abstract

The capability to accurately detect and monitor landslides is essential for understanding their dynamics and reducing associated risks. However, existing deep learning models often struggle to effectively capture temporal dynamics from satellite imagery, limiting their reliability in analyzing landslide behavior over time. To address this limitation, Sen12Landslides is introduced, a large-scale, multi-modal, multi-temporal dataset designed for satellite-based landslide monitoring and spatio-temporal anomaly detection. Sen12Landslides contains 75,000 landslide annotations from 15 diverse regions globally and over 12,000 patches derived from Sentinel-1 SAR, Sentinel-2 optical imagery, and Copernicus DEM. Each patch includes pixel-level annotations and precise event dates with pre- and post-event timestamps. The dataset supports advanced deep learning approaches, capturing spatial features and temporal changes critical for landslide detection. Benchmark experiments using established models, including U-ConvLSTM, 3D-UNet, and U-TAE, demonstrate the dataset’s utility for landslide detection, with the best-performing model achieving an F1-score exceeding 83% on Sentinel-2 data. By providing this comprehensive resource, Sen12Landslides enables more robust model training and promotes generalization across regions, advancing research in Earth observation and geohazard monitoring.

## Background & Summary

Landslides represent a significant global hazard, typified by the downward movement of a mass of soil, rock, or debris under the force of gravity^1^. These events often occur as a consequence of substantial seismic events, extreme weather conditions, such as substantial precipitation or volcanic eruptions, or anthropogenic activities^2,3^. Due to their serious impacts on infrastructure and human life, particularly in mountainous regions, landslides represent a critical global challenge, affecting millions of people and underscoring the urgent need for comprehensive monitoring and mitigation strategies, such as early warning systems^4,5^. In the context of global climate change, the increasing frequency of landslides and the associated risks and damages highlight the need for detailed spatial mapping and systematic documentation in centralized inventory datasets^6^. Such inventories are crucial for improving our understanding of landslide processes and spatio-temporal patterns that are essential for dynamic hazard assessments, mobility studies, and the analysis of temporal variations in landslide frequency^3,7^. Additionally, these datasets are essential for creating susceptibility maps based on static, time-invariant factors, such as topography and lithology. By incorporating dynamic trigger factors such as rainfall and seismic activity, they facilitate landslide hazard assessment. When combined with information on exposed elements, such as infrastructure and population, they support comprehensive risk evaluations. This progression from susceptibility to hazard to risk is fundamental to predictive modeling, early warning systems, and informed land use planning^6,8^. Moreover, these inventories are indispensable for training and validating remote sensing and machine learning models, thus enhancing automated detection and observation capabilities over large regions^3^. By combining practical applications with research insights, landslide inventories serve as a cornerstone for extensive monitoring and risk mitigation strategies. Traditional inventory methods, such as field surveys and manual landslide mapping in satellite imagery, have been the mainstay of these inventories. Although reliable, field surveys are time-consuming and pose significant risks in large or remote areas^9^. Similarly, manual delineation of landslides in satellite imagery is a labor-intensive process that requires significant time, resources, and expertise. This makes the task of creating and updating inventories difficult^10,11^.

The advent and exponential growth of Earth Observation (EO) data, which is expected to increase by tens of petabytes per year due to new satellite launches, along with advances in observational technology and digital image processing, have significantly expanded the use of remote sensing in landslide research and disaster monitoring^12,13^. Remote sensing techniques have emerged as a critical tool in landslide investigations, providing a systematic, extensive, and cost-effective view of the Earth’s surface at different scales^14^. When used in conjunction with advanced machine learning or deep learning methodologies, this synergy greatly enhances the ability for large-scale analysis and frequent inventory updates, especially after trigger events^3,15–17^. This approach not only overcomes many limitations of traditional methods, but also leverages the detailed information provided by remote sensing to better understand landslides, effectively facilitating the creation and updating of landslide inventories^14,18^. When it comes to collecting satellite data for specific landslide inventories, the published datasets are usually based on single or bi-temporal images that capture isolated snapshots in time^19–21^. While these datasets are valuable for creating inventories, their limitations become apparent when training detection models or conducting further analysis. The training of models on this type of data can result in misclassification or misidentification of regular land surface changes as landslides, due to their similar visual or spectral features. For example, riverbeds exposed after heavy rainfall can visually resemble landslides, potentially confusing models and reducing detection accuracy. A model trained on multi-temporal data could learn to distinguish such cases by indentifying patterns, such as the proximity of riverbeds to watercourses and their consistent seasonal changes, that are not typical of landslide events. This highlights the importance of multi-temporal analysis in capturing the dynamic and complex nature of landslide activity, and therefore enhancing the reliability and generalizability of detection models^15,22–24^.

Relying on a single type of sensor for tasks such as monitoring, detection, and inventory poses additional challenges because it often results in incomplete information and misses valuable complementary signals from other modalities^2^. For example, optical remote sensing is widely used but is less effective in adverse weather conditions or areas obscured by clouds or dense vegetation. In contrast, Synthetic Aperture Radar (SAR) technology offers a significant advantage by penetrating such obstructions, facilitating continuous, all-weather ground observations and overcoming some of the critical limitations of optical sensors^25^. However, SAR imagery is inherently more complex and often more difficult to interpret, typically requiring specialized processing techniques and expertise. The incorporation of Digital Elevation Models (DEM) has also been shown to significantly improve landslide detection performance by providing additional topographic information that increases detection accuracy and helps reduce false classifications^26,27^.

In the era of deep learning (DL), annotated datasets such as landslide inventories have become indispensable resources for remote sensing applications, from semantic segmentation to object detection and localisation. These datasets play a central role in training and validating DL algorithms. While noteworthy initiatives, such as Landslide4Sense^19^, have made significant contributions to addressing the need for benchmark datasets, their reliance on four inventories and their use of single-temporal data indicate the necessity for further advancements. Furthermore, the absence of additional geospatial layers (annotations or metadata) limits the dataset’s usefulness for other types of studies, making it difficult to apply beyond its original scope, regardless of the machine learning methods used^19^. Building on the foundation of this work, this paper extend its scope by developing a more comprehensive and diverse dataset, incorporating multi-temporal data enriched with SAR and DEM information, and providing additional geospatial data with extensive metadata to support more complex tasks.

In response to the limitations identified in existing works, Sen12Landslides presents a large-scale, multi-modal, and multi-temporal resource tailored for satellite-based landslide monitoring. The dataset offers a unified and openly accessible inventory of polygon-based landslides, enriched with satellite imagery from Sentinel-1, Sentinel-2, and elevation data from the Copernicus DEM. Spanning 15 diverse regions worldwide, it encompasses detailed metadata, including trigger type, event date, and landslide size, facilitating precise spatial and temporal analysis. The utility of the dataset is demonstrated through semantic segmentation experiments on our satellite image time series (SITS) using state of the art deep learning models such as U-ConvLSTM, 3D-UNet, and U-TAE, achieving accurate and temporally consistent spatial predictions. The dataset’s ability to support spatio-temporal anomaly detection, multi-modal data fusion, and supervised learning tasks makes Sen12Landslides a robust foundation for advancing geohazard analysis. The dataset is made publicly available to foster innovation in remote sensing and machine learning and to promote a deeper understanding of landslide dynamics on a global scale.

## Methods

**Fig. 1:**
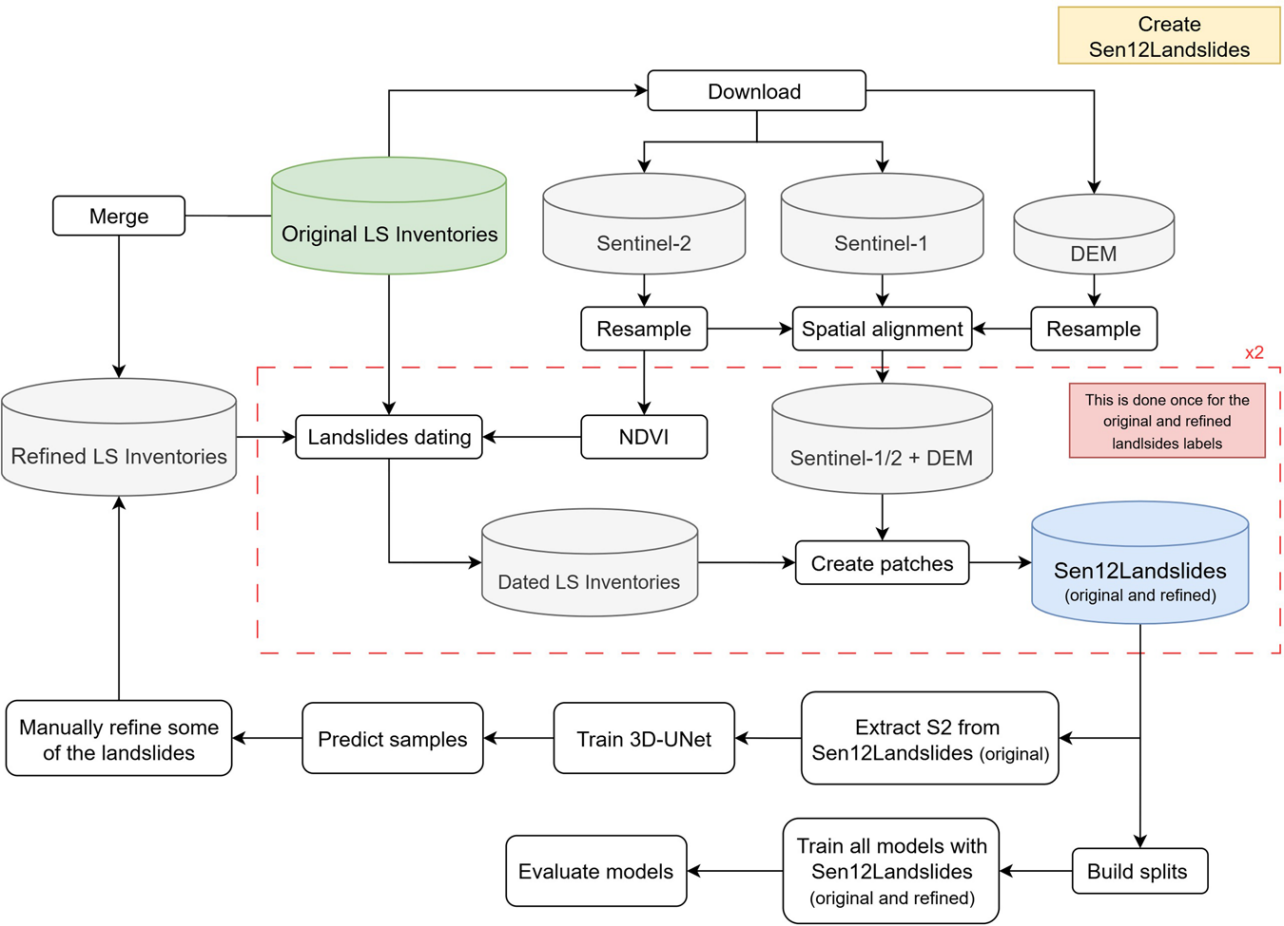
Overview of the Sen12Landslides processing pipeline, integrating landslide inventories with Sentinel-1, Sentinel-2, and DEM data for dataset creation and subsequent deep learning model training.

This chapter outlines the development of the Sen12Landslides dataset. Figure 1 illustrates the overall workflow, detailing each key step in the process.

The construction of Sen12Landslides begins with the integration of existing landslide inventories (“Original LS Inventories”), which guide the acquisition of Sentinel-1 and Sentinel-2 time series and the Copernicus DEM (Fig. 1). The satellite datasets are resampled to 10 m, spatially aligned, and combined with NDVI time series. Using a custom dating algorithm, missing pre-, event-, and post-event dates are inferred and used together with the original polygons to generate patches, that form the first version of the dataset (Sen12Landslides-original). As highlighted in the red box of Fig. 1, the dating and patch creation process is performed twice: first with the original inventories, and again with refined inventories. The refined version is generated by training a deep learning model on Sentinel-2 patches from the original dataset and applying it to regions with annotation gaps. The labels are then manually refined and the corrected annotations are merged with unchanged inventories to produce an updated inventory (“Refined LS Inventories”), from which a second version of the dataset is created (Sen12Landslides-refined). Finally, dataset splits are built for both versions and used to train and evaluate multiple deep learning models, enabling comparison across modalities, label quality, and dataset robustness (Fig. 1). The next sections provide more details about each step.

### Landslide Inventories

**Fig. 2:**
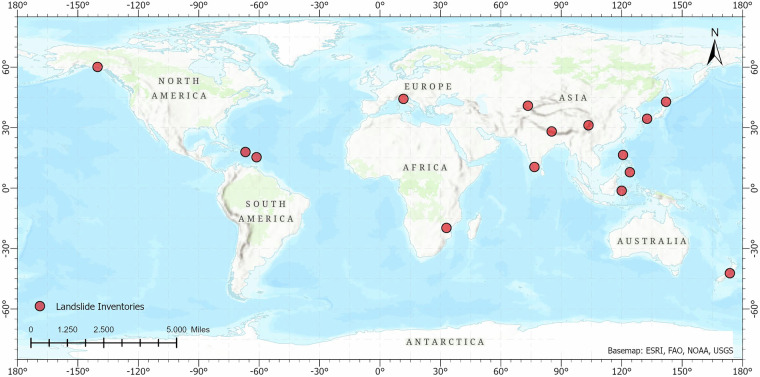
Global distribution of landslide inventories.

The 15 landslide inventories used in this study form a globally distributed and representative dataset, selected based on key quality criteria such as spatial diversity, temporal consistency, relevance, and minimal bias^28^. This global selection ensures broad coverage of different environmental conditions and regional characteristics, capturing both spatial heterogeneity across geographic regions and temporal variability driven by factors such as seasonal changes (Fig. 2). After basic quality checks (e.g., validating polygon geometries, verifying attribute columns), the dataset used in the initial training phase includes 126,086 landslides from 13 countries recorded between 2016 and 2023. The final version, reflecting refined and updated annotations, contains 74,956 landslides. A detailed list of the selected inventories, including their geographic and temporal coverage, is provided in Table 1, with further metadata information outlined in the Data Records section.

**Table 1:** Summary of landslide inventories integrated into the unified database, including authorship, country, event type, temporal coverage, landslide counts (original vs. refined) and references with a link.

Country	Event Type	Landslides (orig.)	Landslides (ref.)	Time Span	Reference	URL
USA	Multi	227	17	2017-01–2019-08	^53^	10.5066/P9FZUX6N
Italy	Single	47,523	47,523	2022-10–2023-09	^11^	Italy Emilia region landslides
Zimbabwe	Single	1,320	5,042	2018-09–2019-09	^54^	10.5194/nhess-22-1129-2022-supplement
China	Multi	1,101	3,125	2016-07–2022-01	^15^	GitHub Repo
Dominica	Single	10,552	9,168	2017-02–2018-05	^54^	10.5194/nhess-22-1129-2022-supplement
Nepal	Multi	54	325	2016-12–2020-04	^15^	GitHub Repo
Japan	Single	2,018	5,023	2017-12–2019-01	^54^	10.5194/nhess-22-1129-2022-supplement
Japan	Single	135	3,569	2018-02–2019-03	^55^	GSI Japan
Philippines	Single	458	698	2018-03–2019-03	^54^	10.5194/nhess-22-1129-2022-supplement
New Zealand	Multi	50,658	21,268	2016-03–2022-05	^15^	GitHub Repo
Philippines	Single	17	358	2017-06–2018-08	^54^	10.5194/nhess-22-1129-2022-supplement
Kyrgyzstan	Multi	7,955	7,358	2015-12–2019-12	^4^	GFZ Potsdam
USA	Multi	303	568	2019-07–2020-09	^53^	10.5066/P9FZUX6N
Indonesia	Multi	3,378	1,374	2016-02–2019-12	^56,57^	Global Landslide Catalog
India	Single	417	814	2017-11–2019-03	^54^	10.5194/nhess-22-1129-2022-supplement

### Sentinel-1/-2 and DEM

Sentinel-1A and Sentinel-1B are satellites that provide C-band dual-polarization (VV and VH) SAR imagery, systematically mapping most of the world’s landmasses every 12 days and European land and coastal waters every six days in both ascending and descending passes^29^. For the present study, Normalized Radar Backscatter (NRB) products derived from Interferometric Wide Swath (IW) acquisitions were collected, including both polarisations (VV and VH) for ascending and descending orbits^30^. The application of such radiometric terrain-corrected products is critical for landslide analysis in mountainous regions. The data selection spans one year before and after each identified landslide trigger event, to assure complete coverage.

Sentinel-2A and Sentinel-2B provide high-resolution optical imagery (10-60 meters) in 13 spectral bands, covering terrestrial and coastal areas with a swath of 290 km. Their combined operation improves the revisit interval from 10 to 5 days^31^. Acquisitions were filtered similar to Sentinel-1 to cover one year before and after each landslide trigger event. Due to low information content all Sentinel-2 bands with a spatial resolution of 60 meters were excluded, resulting in 10 remaining spectral bands.

The Copernicus Digital Surface Model (DSM) is a digital representation of the Earth’s surface, including features such as buildings, vegetation, and infrastructure. This model is available in various spatial resolutions to support a wide range of applications. In this study, the GLO-30 instance was utilized, providing global coverage at 30-meter resolution^32^. The integration of the Copernicus DEM provides valuable slope and elevation information, which is essential for landslide mapping due to the strong correlation between topography and landslide occurrence and movement^27^. Recent research highlights the benefits of combining Sentinel-2 data with slope and elevation information to improve detection accuracy, including approaches using multi-modal data and advanced DL models^19,33^.

All Sentinel-1 and Copernicus DEM data were accessed via terrabyte, a high-performance data analytics (HPDA) platform developed by the German Aerospace Center (DLR) and the Leibniz Supercomputing Centre (LRZ). Sentinel-2-L2A imagery was retrieved from the Microsoft Planetary Computer^34^.

In accordance with the workflow delineated in Fig. 1, the ingested Sentinel-1, Sentinel-2, and DEM data were processed through a resampling procedure, resulting in a uniform 10-meter resolution. Subsequently, the data were clipped to a common spatial extent based on the Sentinel-2 tile grid, covering all inventoried landslides. Instead of fusing the data along the time dimension, the original timestamps of the Sentinel-1 and Sentinel-2 images were preserved. This retains critical time series information that is essential for anomaly detection tasks that require precise temporal alignment. The resulting dataset, described as “Sentinel-1/2 + DEM” in Fig. 1, consists of raster images of consistent spatial resolution and geographic extent for each time stamp within the Sentinel-1 and Sentinel-2 time series, complemented by terrain data from the Copernicus DEM. It is important to note that the DEM is static and only represents a single point in time.

### Landslide Dating

Following previous studies^35,36^, an existing framework that integrates optical and SAR remote sensing was adopted to date landslides. This approach assumes that landslides result in vegetation loss, which can be detected through changes in the Normalized Difference Vegetation Index (NDVI), and physical surface changes, observable via SAR coherence and backscatter (VV and VH)^35,36^. However, our analysis revealed that Sentinel-1 data exhibited substantial noise, with minimal changes in VV and VH time series associated with landslides. In contrast, NDVI time series consistently showed clear patterns of vegetation loss over affected areas, confirming NDVI as a more reliable indicator for landslide timing. As a result, the framework was refined to prioritize NDVI analysis and to simplify the detection of landslide events.

**Fig. 3:**
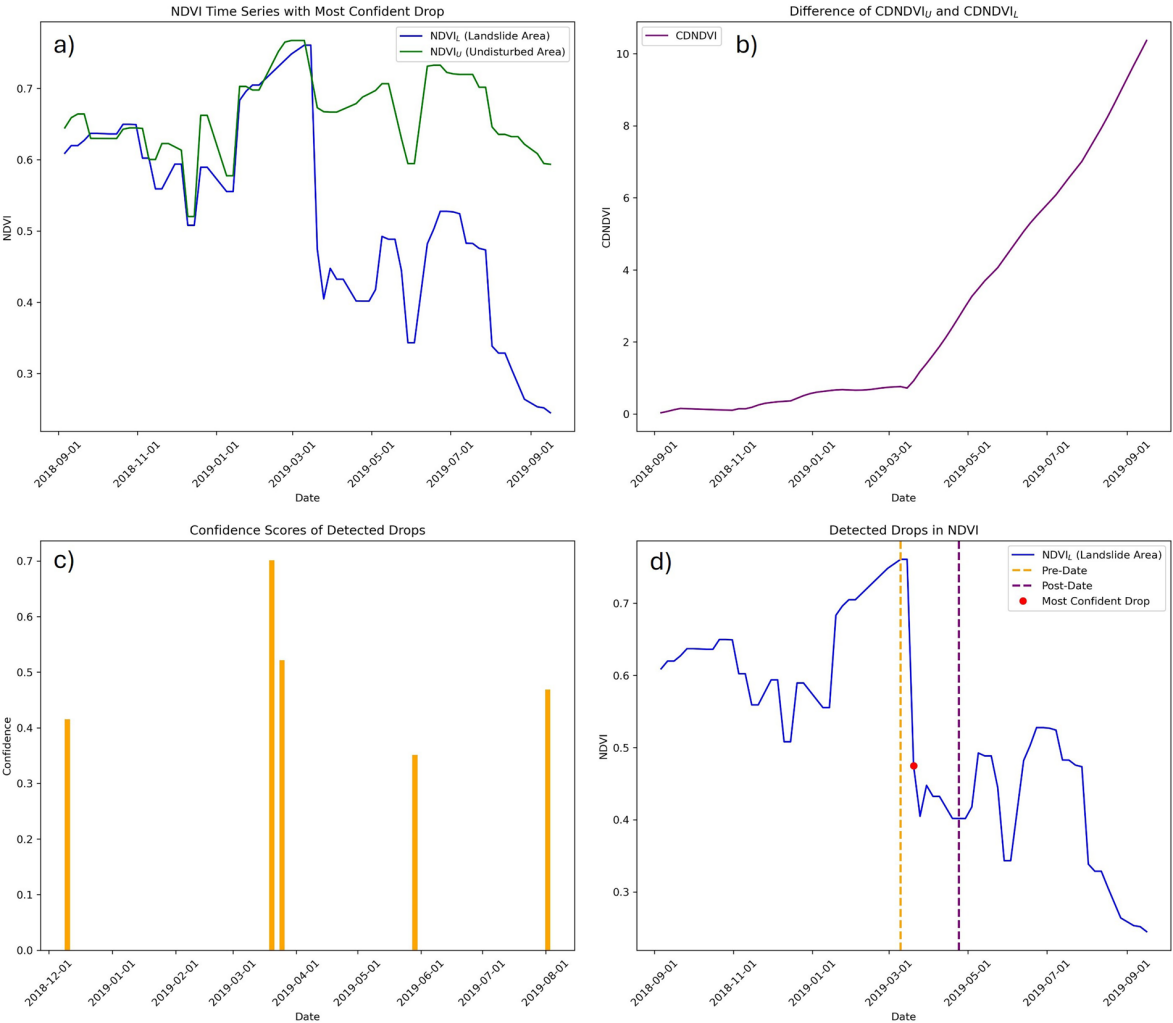
Workflow for NDVI-based landslide event dating: (**a**) NDVI time series comparison between landslide and undisturbed areas; (**b**) cumulative difference between the undisturbed NDVI and the landslide NDVI (CDNDVI); (**c**) confidence scores for detected NDVI drops; and (**d**) identified landslide date, including pre- and post-event windows with the highest-confidence drop highlighted.

For this purpose, mean NDVI values were calculated from landslide-affected areas (NDVI_*L*_) and from undisturbed vegetation within a 300 m buffer (NDVI_*U*_), carefully excluding any other landslides within the buffer, as they often occur in close proximity^35,36^. The cumulative NDVI difference, defined as the deviation between the observed NDVI and a fitted harmonic model, was then computed for both landslide and undisturbed areas.^37^. The difference between these two results is shown in the upper right corner of Fig. 3 and should indicate the timing of the landslide. However, when applied across diverse conditions in other inventories, this method proved inconsistent due to residual noise in the signal, and in many cases, clear pre- and post-event periods could not be reliably identified. Consequently, the analysis primarily focused on the raw NDVI_*L*_ and NDVI_*U*_ time series to assess vegetation loss and estimate landslide occurrence dates.

After collecting all the NDVI_*L*_ and NDVI_*U*_ data for each landslide, the analysis focused on identifying significant drops in NDVI_*L*_ values. A drop was defined as a decrease greater than 0.2 between two consecutive time steps. Given the potential for multiple such drops within a single landslide time series, a confidence score was calculated for each detected drop to identify the most probable landslide event. It ranges between 0 and 1, where a value of 1 indicates high confidence in the date and values closer to 0 indicate lower confidence (e.g., due to noisy data.

The confidence score *C* is calculated as:

$$C=0.4\left(\frac{{D}_{{\rm{below}}}}{N}\right)+0.4\,{P}_{{\rm{post}}}+0.2\left(\frac{M}{{M}_{{\rm{range}}}}\right)$$

where:

- *D*_below_ is the number of consecutive time steps after the drop during which NDVI_*L*_ < NDVI_*U*_,
- *N* is the total number of time steps in the NDVI time series,
- *P*_post_ is the proportion of NDVI_*L*_ values within a one-year post-drop window that remain below the pre-drop median, calculated from the one-year period before the drop,
- *M* = NDVI_*L*_[*t*] − NDVI_*L*_[*t* + 1] is the magnitude of the drop, measured directly between two consecutive time steps, and
- ${M}_{{\rm{range}}}=\max ({{\rm{NDVI}}}_{L})-\min ({{\rm{NDVI}}}_{L})$ is the maximum observed drop used for normalization.

The confidence score combines three key aspects: (1) the relative duration during which NDVI_*L*_ remains below NDVI_*U*_ (representing the temporal extent of the event), (2) the persistence of the drop throughout the one-year post-event period (indicating signal stability), and (3) the magnitude of the drop itself (reflecting the severity of the event), normalized by the overall NDVI range. These components are weighted at 40% for duration, 40% for persistence, and 20% for magnitude, reflecting their relative importance in identifying the most likely landslide event. A visual example of detected drops and their corresponding confidence scores is shown in the lower left panel of Fig. 3, based on a sample landslide from the Zimbabwean inventory. The drop with the highest score is selected as the most probable landslide occurrence.

In order to define the temporal extent of the selected event, a window of ten time steps before and after the detected drop is considered. The pre-event date is identified as the point of highest NDVI within the pre-event window, representing peak vegetation prior to the decline, while the post-event date corresponds to the lowest NDVI value in the post-event window, indicating maximum disturbance. These dates, along with the event date, are added to the landslide inventory and shown in the bottom right panel of Figure 3. This approach ensures precise temporal localisation while accounting for vegetation variability and recovery dynamics.

If an event date is provided in the original landslide inventory, typically based on field reports or media sources, it is considered more reliable than automatically determined dates and is used directly. In these cases, the pre- and post-event periods are defined as seven days before and after the known event date. This fixed window is sufficient for subsequent analysis steps, such as selecting satellite imagery that captures conditions surrounding the event. When a known event date is used, the confidence value is set to 1 to reflect its high reliability. Giving priority to known event dates improves overall data quality and consistency, which is especially important for anomaly detection tasks where both the occurrence and timing of the event must be accurately identified.

The implementation of such a framework in a diverse dataset of approximately 75,000 landslides presents significant challenges. Real-world conditions frequently differ from ideal scenarios due to factors such as data gaps caused by cloud cover, lack of suitable undisturbed reference vegetation, short vegetation cycles, or environmental influences such as snow cover. Therefore, not all automatically assigned dates are perfectly accurate. In such cases, the confidence score gives a valuable metric for assessing the reliability of detected events and can be used to filter uncertain results depending on the specific requirements of downstream applications. After the dating process, each landslide is assigned a pre-event, event, and post-event date. This classification is essential for the selection of corresponding satellite imagery.

### Building Sen12Landslides

As shown in Fig. 1, once the landslide dating algorithm has been applied, the next step is to construct the multi-temporal image patches that form the core of the Sen12Landslides dataset. This process integrates the landslide inventories with the temporally and spatially processed satellite imagery. Given the differences in temporal resolution and acquisition characteristics between the datasets, separate patches are created for Sentinel-1 ascending, Sentinel-1 descending, and Sentinel-2 imagery. This separation ensures internal temporal consistency within each subset.

The first step in constructing the Sen12Landslides dataset was to divide each inventory area into a uniform grid of 128 × 128 pixel cells, without an overlap between adjacent cells. Each grid cell was then treated as a potential patch, and multi-temporal data from Sentinel-1, Sentinel-2, and the Copernicus DEM were assembled for each one. All grid cells intersecting at least one landslide polygon were selected, and if possible an equal number of non-annotated grid cells were randomly chosen to balance the dataset. This process yielded most of the times a 50/50 ratio of annotated (landslide) and non-annotated (non-landslide) patches, thereby assuring a balanced representation for model training and evaluation.

For each designated patch, all available satellite images within the corresponding time span, as defined in Table 1, were retrieved. To ensure consistent temporal coverage across the dataset, the full time span for each patch was divided into 15 equal intervals, regardless of whether the patch contained landslide annotations. From each interval, the best available image was selected based on data quality, using cloud cover for Sentinel-2 and the fraction of invalid pixels for Sentinel-1. This approach enabled a uniform and high-quality time series across all patches. For inventories with a single triggering event, this resulted in high temporal density due to the shorter time horizon. In contrast, inventories with multiple triggering events required a longer observation period, which led to larger gaps between time steps. Nevertheless, all landslide events were included in the selected time series, with at least one image present both before and after each event.

This approach standardized the time series of each patch to 15 images, with the objective of balancing event coverage, temporal consistency, and data quality. Despite these efforts, Sentinel-1 data posed significant challenges due to frequent gaps and regions with invalid or missing observations. The limited availability of usable Sentinel-1 data, particularly for specific orbit directions or acquisition dates, sometimes prevented the creation of complete time series patches. As a consequence, the number of patches across the different data modalities was uneven (see Data Records).

The implementation of a unified NetCDF file for each final patch served to optimize downstream machine learning operations. This file contained the Sentinel-1 or Sentinel-2 time series, the digital elevation model (DEM), and a binary landslide mask. This mask encodes landslide-affected pixels as one and unaffected pixels as zero, ensuring perfect spatial alignment across all image bands, the DEM, and the annotation layers. Each patch is accompanied by extensive metadata, including a unique landslide identifier, the event date, indices of the pre- and post-event images within the time series, and the bounding box geometry of each landslide. This metadata facilitates precise temporal anomaly detection and provides detailed information about the occurrence of each landslide within the sequence. The central latitude and longitude of each patch, the coordinate reference system (CRS), and a flag indicating the presence of annotated landslides are also included. Detailed information can be found in the next chapter (Data Records).

**Fig. 4:**
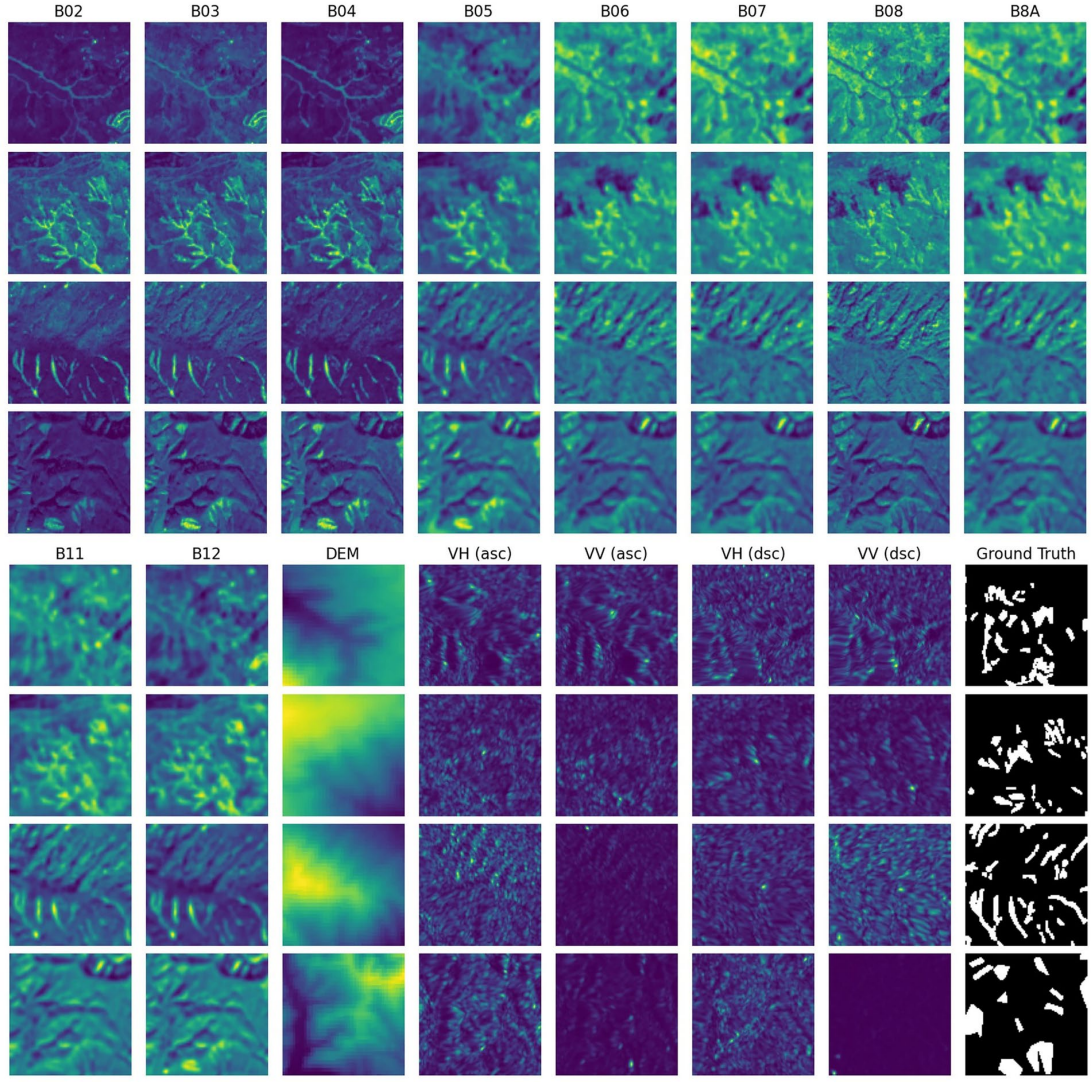
Example of Sen12Landslides input patch composition at a single timestep, showing Sentinel-2 spectral bands (B02-B12), Sentinel-1 backscatter bands (ascending and descending orbit, VV and VH), digital elevation model (DEM), and the binary ground truth mask indicating landslide areas.

As illustrated in Fig. 4, each row shows one example patch from the Sen12Landslides dataset at a single time step, combining all relevant input and target data. The columns begin with the 10 optical Sentinel-2 bands, followed by DEM, the VH and VV polarizations from Sentinel-1 ascending, then those from Sentinel-1 descending, and finally the binary ground truth mask. Although Sentinel-1 and Sentinel-2 data are processed as separate multi-temporal patches, they are here combined for visualization purposes only. This side-by-side view confirms spatial alignment across all modalities and highlights the rich combination of spectral, radar, and topographic information available for each patch.

The result of this data preparation process is the initial version of the Sen12Landslides dataset, constructed using the original landslide annotations. As can be seen in Fig. 1, the subsequent step involved using this dataset to train deep learning models, with the training procedure described in detail in the Technical Validation section. The visual predictions and initial performance metrics indicated that the ground truth data was noisy, particularly in inventories derived from deep learning models. In some cases, landslides visible in the imagery were absent from the annotations, while in others, mountainous areas with minimal or no vegetation were erroneously labeled as landslides. This is likely due to the challenges of large-scale manual mapping, time constraints, the use of machine-generated annotations, and the visual similarity between certain terrain types and landslide features in satellite imagery. Therefore, a refinement process was applied, drawing inspiration from the concept of self-training. In this approach, a trained model is utilized to generate pseudo-labels for unlabeled samples. These pseudo-labels are then refined and converted into labelled training data based on specific quality criteria^28,38^. This approach is in line with recommendations in the literature, where coupling manual interpretation and intervention with automated approaches has been suggested as a promising solution to improve the quality of detected landslides and generated inventories^15^.

Employing the trained models and their predictions, a subset of landslides across the inventories was manually refined based on strict quality criteria. In addition, to increase the reliability of the labels, the available digital elevation model (DEM) was used to exclude landslides located in flat terrain (slope < 7%^24^), as such areas are unlikely to experience landslides and often result in false positives^39^. Additional checks were performed using the high-resolution basemaps in QGIS^40^ and our downloaded post event Sentinel-2 imagery. Subsequent to this refinement, the event-centric process previously outlined was repeated (see red box in Fig. 1). This process included determining event dates, assigning pre- and post-event time steps to each updated landslide, and generating new patches. This iterative process culminated in the final, refined version of the Sen12Landslides dataset, which was then used next to the dataset version with the original (unrefined) annotations for evaluation in the Results section.

## Data Records

The Sen12Landslides dataset (refined version) is available at HuggingFace^41^ and consists of a collection of NetCDF files, each representing a spatio-temporal patch composed of multi-sensor satellite imagery, a digital elevation model (DEM), binary landslide masks, and relevant metadata. To reduce the number of individual files and speed up transfers, multiple patches are packed into compressed tar archives (.tar.gz). A comprehensive README file is included, that describes the structure of the dataset, the contents of the file, and provides instructions on how to download, use or customize the dataset for various tasks and domain-specific settings.

Each patch represents a fixed spatial extent of 128  × 128 pixels over a time series of 15 images. Patches contain either Sentinel-2 or Sentinel-1 imagery, aligned across the spatial grid and temporally sampled at regular intervals. Sentinel-2 patches consist of 10 spectral bands (B02-B12), the scene classification layer (SCL), a binary landslide mask (MASK), and the digital elevation model (DEM). Sentinel-1 patches contain two radar backscatter bands (VV and VH) along with the landslide mask and the DEM (Fig. 4). Note that the Sentinel-1 backscatter values have not been converted to decibel (dB) scale, and for Sentinel-2, the recommended reflectance offset correction for data acquired after January 2022^42^ has not been applied. However, users can easily apply these adjustments using the preprocessing functions provided in the accompanying GitHub repository (see section Code Availability).

Each file also contains metadata attributes that provide additional context for downstream use. In cases where multiple landslides are present within a patch, these attributes are stored as lists, with each entry corresponding to a specific landslide. The metadata includes landslide annotation identifier (ann_id), their bounding boxes (ann_bbox), estimated event dates (event_date), and confidence scores for those dates (date_confidence). Temporal information is provided as indices of the pre- and post-event images within the time series (pre_post_dates). The ‘annotated` flag indicates whether at least one landslide is present, and the satellite source is recorded under ‘satellite‘. Geolocation details such as the center latitude and longitude of the patch (center_lat, center_lon) and its coordinate reference system (crs) are also included.

The dataset contains a total of 13,628 Sentinel-2 patches, 13,306 Sentinel-1 ascending orbit patches (S1-asc), and 12,622 Sentinel-1 descending orbit patches (S1-dsc). In addition to the Sen12Landslides dataset, two predefined and task-specific versions are provided, both derived from the same set of image patches. The S12LS-LD version is tailored for landslide detection tasks and features a higher ratio of annotated to unannotated samples (80:20), while the S12LS-AD version is designed for anomaly detection and includes only patches with high-confidence (1.0) event dates, using a balanced 50:50 ratio. These variations enable targeted experimentation across different use cases. For maximum flexibility, users can also generate customized versions of the dataset using the provided script, allowing adaptation to specific research questions or application domains.

In addition to the patch data, an updated landslide inventory is provided with detailed metadata for each annotated event. The metadata includes an identifier, pre- and post-event dates, event date, confidence score of the dating process, triggering event, affected area, location, landslide type with velocity class (based on^43^), contributing authors, and a reference link for downloading the data. Table 2 presents a sample of selected entries from the global inventory, illustrating the structure of the database. This standardized inventory can also serve as a reference for users who wish to explore individual landslide events more dynamically, for example by linking to external data sources or performing case-specific analyses in applications such as QGIS^40^.

**Table 2:** Example metadata structure for landslide inventories, including event dates with confidence score, triggering factors, affected area, landslide types, authorship, and reference.

Id	Pre Date	Event Date	Post Date	Confidence	Trigger	Area (m^2^)	Location	Type	Velocity	Author	Reference
0	2019-03-08	2019-03-15	2019-03-22	1.0	Rainfall	10163.6	Chimanimani	Debris Flow	≥5	Höhn, P. *et al*.^41^	DOI
...	...	...	...	...	...	...	...	...	...	...	...
13700	2018-06-21	2018-06-28	2018-07-05	1.0	Rainfall	2842.7	Hiroshima	Debris Flow	≥5	Höhn, P. *et al*.^41^	DOI
...	...	...	...	...	...	...	...	...	...	...	...
55774	2023-05-09	2023-05-16	2023-05-23	1.0	Rainfall	1772.3	Italy	Debris Flow	≥5	Ferrario, M. F. *et al*.^11^	Zenodo
...	...	...	...	...	...	...	...	...	...	...	...
69004	2017-06-08	2017-06-28	2017-09-16	0.67	—	71049.7	Kyrgyzstan1	Debris Flow	≥5	Höhn, P. *et al*.^41^	DOI
...	...	...	...	...	...	...	...	...	...	...	...
74955	2019-12-31	2020-01-07	2020-01-14	1.0	Earthquake	282.1	PuertoRico	Debris Flow	≥5	Belair, G. M. *et al*.^53^	DOI

**Fig. 5:**
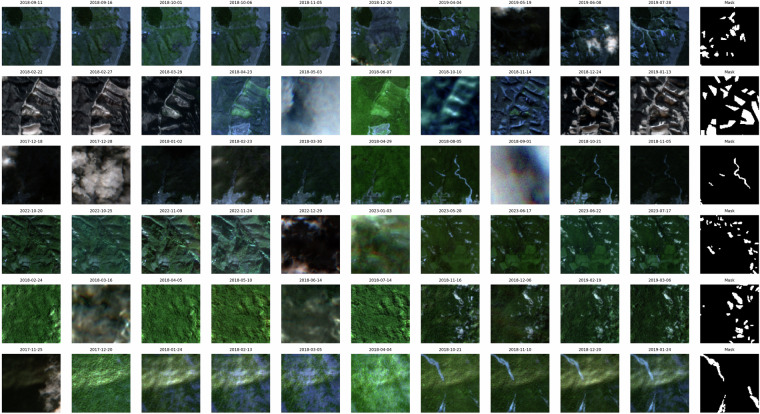
RGB composites of Sentinel-2 patches at multiple timesteps, illustrating temporal variability, terrain conditions, and clearly visible landslide anomalies.

In order to provide a visual representation of the selected patches of Sen12Landslides, RGB images of the Sentinel-2 patches rendered over selected representative time steps are presented. These visualizations are displayed in Fig. 5 and highlight the progression of events as well as the variety of conditions under which landslides and anomalies must be detected.

## Technical Validation

### Implementation Details

The Sen12Landslides dataset was evaluated using a selection of robust deep learning models that have been successfully applied to semantic segmentation of SITS in related domains. The selected models include ConvGRU^44,45^, U-ConvLSTM^46^, 3D-UNet^46^, and U-TAE^47^. These architectures are designed to capture both spatial and temporal dependencies and were selected to evaluate the ability of the dataset to support spatio-temporal feature learning for landslide detection.

Three independent runs were conducted using stratified data splits for reproducibility and easier analysis. Class imbalance, a common challenge in landslide detection^33^, was addressed by classifying patches into three classes based on landslide coverage (ratio of landslide to total pixels) using the 25th and 75th percentiles. Patches below the 25th percentile were assigned to class 0, between the 25th and 75th to class 1, and above the 75th to class 2. These classes guided the stratified splitting, ensuring balanced representation across training, validation, and test sets (8:2 split, with training further split 8:2). Similar stratified sampling strategies have shown benefits in remote sensing tasks^48^.

A fair comparison across modalities (Sentinel-1 ascending, Sentinel-1 descending, Sentinel-2) and label versions (original vs. refined) was ensured by using a unified test set for all experiments. Models were trained independently for each modality, and predictions for Sentinel-1 ascending and descending orbits were merged into a single Sentinel-1 result, as reported in the metrics tables. The same approach was applied to combine Sentinel-1 and Sentinel-2 predictions (Sentinel-1/2). This merging strategy was chosen as the simplest and most practical solution, even though direct stacking is possible despite differing temporal resolutions in the multi-modal SITS^49^. Data normalization was performed separately for each run, using statistics from the corresponding training subset to maintain consistency and prevent information leakage.

Training was conducted on the joint HPDA infrastructure terrabyte of the German Aerospace Center (DLR) and the Leibniz Supercomputing Center (LRZ) using NVIDIA A100-SXM4-80GB GPUs. Models were trained on a single GPU with batch sizes of 32 (U-TAE, 3D-UNet, ConvGRU) and 24 (U-ConvLSTM). Across three runs, training took 6-8 h for U-TAE, 3D-UNet, and ConvGRU, and 19-22 h for U-ConvLSTM. All models trained for 50 epochs, as longer runs showed no performance gains. The setup used Adam optimizer, cross-entropy loss, an initial learning rate of 10^−4^, and a scheduler reducing the rate upon validation plateau.

Following previous works^2,19,33^, model performance was evaluated using precision, recall, and F1 score, calculated on the test set of each independent run. The final scores were averaged across the three runs to ensure a robust and reproducible evaluation. This evaluation is intended as a proof-of-concept to demonstrate the practical feasibility of using the Sen12Landslides dataset for semantic segmentation tasks, rather than to establish new state-of-the-art performance benchmarks.

### Experiments

This section presents experiments on the Sen12Landslides dataset to validate its technical quality, demonstrate its practical relevance for semantic segmentation in landslide detection, and highlight key features.

#### Experiment 1

The objective of the first experiment is to assess whether the incorporation of refined labels enhances model performance in comparison to the original annotations. All models were trained separately on both the original and refined datasets, while being tested on the same set of patches. Table 3 presents the outcomes for Sentinel-1 (S1), Sentinel-2 (S2), and the combined Sentinel-1/2 (S1/2) data. Across all models and both modalities, it was observed that F1 scores were consistently higher when trained on refined labels, with improvements ranging from 4-7%. For example, on Sentinel-2 data, U-ConvLSTM improved its F1 score from 0.78 to 0.83, while 3D-UNet increased from 0.77 to 0.80. This consistent gain underscores the efficacy of the manual label refinement process, which yielded more accurate and less noisy supervision signals for training. Based on these findings, the refined dataset was used in all other experiments to have an optimal training signal.

**Table 3:** Model performance metrics (Precision / Recall / F1  ± std) using original versus refined labels on Sentinel-1, Sentinel-2, and combined Sentinel-1/2 datasets. Best F1 scores for each modality are highlighted in bold.

Model	Original (P/R/F1)	Refined (P/R/F1)
**Sentinel-1 (S1)**		
ConvGRU	0.81 ± 0.01 / 0.54 ± 0.00 / 0.57 ± 0.00	0.83 ± 0.02 / 0.58 ± 0.01 / 0.63 ± 0.01
U-ConvLSTM	0.77 ± 0.01 / 0.58 ± 0.02 / 0.63 ± 0.01	0.81 ± 0.01 / 0.64 ± 0.01 / **0.69 ± 0.01**
3D-UNet	0.77 ± 0.02 / 0.59 ± 0.02 / 0.62 ± 0.02	0.83 ± 0.01 / 0.64 ± 0.02 / **0.69 ± 0.01**
U-TAE	0.75 ± 0.03 / 0.58 ± 0.02 / 0.62 ± 0.01	0.79 ± 0.02 / 0.62 ± 0.01 / 0.66 ± 0.01
**Sentinel-2 (S2)**		
ConvGRU	0.83 ± 0.02 / 0.66 ± 0.01 / 0.72 ± 0.01	0.83 ± 0.00 / 0.66 ± 0.01 / 0.72 ± 0.01
U-ConvLSTM	0.86 ± 0.01 / 0.72 ± 0.00 / 0.78 ± 0.01	0.87 ± 0.01 / 0.79 ± 0.01 / **0.83 ± 0.01**
3D-UNet	0.85 ± 0.01 / 0.72 ± 0.01 / 0.77 ± 0.00	0.87 ± 0.01 / 0.76 ± 0.03 / 0.80 ± 0.02
U-TAE	0.88 ± 0.00 / 0.69 ± 0.01 / 0.76 ± 0.01	0.90 ± 0.01 / 0.73 ± 0.01 / 0.79 ± 0.01
**Sentinel-1/2 (S1/2)**		
ConvGRU	0.81 ± 0.02 / 0.67 ± 0.02 / 0.72 ± 0.01	0.81 ± 0.01 / 0.69 ± 0.01 / 0.73 ± 0.01
U-ConvLSTM	0.82 ± 0.01 / 0.74 ± 0.00 / 0.77 ± 0.00	0.83 ± 0.01 / 0.81 ± 0.01 / **0.82 ± 0.00**
3D-UNet	0.81 ± 0.02 / 0.74 ± 0.01 / 0.77 ± 0.00	0.83 ± 0.00 / 0.78 ± 0.03 / 0.80 ± 0.02
U-TAE	0.81 ± 0.03 / 0.71 ± 0.01 / 0.75 ± 0.01	0.83 ± 0.01 / 0.75 ± 0.01 / 0.79 ± 0.01

All models across both datasets consistently exhibit higher precision than recall. In the context of landslide detection, this indicates that while the model frequently predicts landslides accurately (high precision), it may occasionally overlook actual landslide areas (low recall). This is a common challenge in unbalanced datasets, where the landslide class is underrepresented. While high precision is advantageous in preventing false alarms, enhancing recall remains crucial to minimize missed detections. The F1 score, a metric that balances precision and recall, serves as a comprehensive metric for evaluating model performance^50^.

#### Experiment 2

The second experiment examines the impact of different data modalities on landslide detection performance. The modalities considered include Sentinel-1, Sentinel-2, and their combination. Table 4 summarizes the results, using refined labels for improved clarity. Across all input types, U-ConvLSTM and 3D-UNet achieve the highest F1 scores, reaching up to 0.83 on Sentinel-2 and 0.82 on combined S1/2 data. U-TAE follows closely, with F1 scores up to 0.79. In contrast, ConvGRU consistently yields the lowest F1 scores across all modalities, highlighting its limited effectiveness in this context. This performance ranking remains stable across S1, S2, and S1/2 inputs.

**Table 4:** Performance comparison (Precision / Recall / F1-Score) of models trained on Sentinel-1 (S1), Sentinel-2 (S2), and combined Sentinel-1/2 (S1/2) datasets using refined labels. Best F1 scores for each model across all modalities are highlighted in bold.

Model	S1	S2	S1/2
	(P/R/F1)	(P/R/F1)	(P/R/F1)
ConvGRU	0.83 ± 0.02 / 0.58 ± 0.01 / 0.63 ± 0.01	0.83 ± 0.00 / 0.66 ± 0.01 / 0.72 ± 0.01	0.81 ± 0.01 / 0.69 ± 0.01 / **0.73 ± 0.01**
U-ConvLSTM	0.81 ± 0.01 / 0.64 ± 0.01 / 0.69 ± 0.01	0.87 ± 0.01 / 0.79 ± 0.01 / **0.83 ± 0.01**	0.83 ± 0.01 / 0.81 ± 0.01 / 0.82 ± 0.00
3D-UNet	0.83 ± 0.01 / 0.64 ± 0.02 / 0.69 ± 0.01	0.87 ± 0.01 / 0.76 ± 0.03 / **0.80 ± 0.02**	0.83 ± 0.00 / 0.78 ± 0.03 / **0.80 ± 0.02**
U-TAE	0.79 ± 0.02 / 0.62 ± 0.01 / 0.66 ± 0.01	0.90 ± 0.01 / 0.73 ± 0.01 / **0.79 ± 0.01**	0.83 ± 0.01 / 0.75 ± 0.01 / **0.79 ± 0.01**

Furthermore, the analysis suggests that Sentinel-2 generally outperforms Sentinel-1 across all models, thereby validating prior research findings that optical data provides stronger cues for identifying landslides^51^. The integration of Sentinel-1 and Sentinel-2 data has been observed to result in an enhancement of recall, accompanied by a marginal decline in precision. This results in only a marginal improvement, or in some cases a plateau, in overall F1 score compared to using Sentinel-2 alone. However, the combined modality provides a more balanced precision-recall trade-off, which can be beneficial for reducing both false positives and missed detections.

This observation stands in contrast to the findings reported in a previous study^2^, where the integration of SAR, DEM, and optical data within a multi-channel U-Net architecture was found to yield substantial performance enhancements. The observed discrepancy can be attributed to the training methodology of this paper, where models are trained independently on each data source and predictions are merged post hoc. This strategy imposes limitations on the model’s capacity to learn shared feature representations across different data modalities. Nevertheless, the incorporation of diverse data modalities establishes a substantial foundation for the further development of integrated, multi-temporal fusion models that will integrate all data sources within a unified end-to-end architecture.

The results in Table 5 present a comparison of model performance when trained on datasets with and without DEM information across S1, S2, and S1/2 inputs. For all data sources and architectures, it is observed that removing the DEM input leads to a consistent decrease in recall, while precision remains largely stable. This leads to only minor differences in the overall F1 score, indicating that the incorporation of DEM in its raw state does not significantly boost model performance during the training process. One possible explanation is that the DEM was used in its unprocessed form without deriving informative terrain features such as slope or aspect, which are more directly relevant to landslide susceptibility. However, the inclusion of DEM in the dataset remains highly valuable as it provides critical topographic context that can be effectively used in pre- or post-processing workflows. For instance, the utilization of DEM data enables the filtration of false positives in areas characterized by flat topography or the refinement of predictions in topographically complex regions, such as mountains.

**Table 5:** Impact of DEM input on model performance (Precision / Recall / F1) for Sentinel-1 (S1), Sentinel-2 (S2), and combined Sentinel-1/2 (S1/2) datasets. Best F1 scores for each model are highlighted in bold.

Model	Dataset	With DEM (P/R/F1)	Without DEM (P/R/F1)
3D-UNet	S1	0.78 ± 0.02 / 0.64 ± 0.01 / 0.69 ± 0.01	0.77 ± 0.02 / 0.63 ± 0.01 / 0.68 ± 0.00
	S2	0.85 ± 0.02 / 0.74 ± 0.01 / **0.79 ± 0.01**	0.84 ± 0.01 / 0.74 ± 0.01 / 0.78 ± 0.01
	S1/2	0.79 ± 0.02 / 0.78 ± 0.01 / **0.79 ± 0.01**	0.78 ± 0.01 / 0.77 ± 0.01 / 0.78 ± 0.00
ConvGRU	S1	0.79 ± 0.01 / 0.58 ± 0.01 / 0.62 ± 0.01	0.80 ± 0.01 / 0.54 ± 0.00 / 0.57 ± 0.00
	S2	0.76 ± 0.01 / 0.66 ± 0.01 / 0.70 ± 0.00	0.79 ± 0.02 / 0.65 ± 0.01 / 0.69 ± 0.01
	S1/2	0.75 ± 0.01 / 0.69 ± 0.01 / **0.71 ± 0.01**	0.78 ± 0.02 / 0.66 ± 0.01 / 0.70 ± 0.01
U-TAE	S1	0.74 ± 0.01 / 0.61 ± 0.01 / 0.65 ± 0.01	0.77 ± 0.02 / 0.59 ± .01 / 0.64 ± 0.02
	S2	0.87 ± 0.01 / 0.72 ± 0.03 / **0.78 ± 0.02**	0.87 ± 0.01 / 0.72 ± 0.02 / **0.78 ± 0.01**
	S1/2	0.79 ± 0.01 / 0.75 ± 0.03 / 0.77 ± 0.01	0.81 ± 0.00 / 0.74 ± 0.02 / 0.77 ± 0.02
U-ConvLSTM	S1	0.75 ± 0.01 / 0.65 ± 0.01 / 0.68 ± 0.01	0.75 ± 0.01 / 0.63 ± 0.02 / 0.67 ± 0.01
	S2	0.85 ± 0.01 / 0.77 ± 0.02 / **0.81 ± 0.01**	0.85 ± 0.01 / 0.76 ± 0.01 / 0.80 ± 0.01
	S1/2	0.78 ± 0.01 / 0.81 ± 0.01 / 0.79 ± 0.01	0.79 ± 0.01 / 0.78 ± 0.01 / 0.78 ± 0.01

#### Experiment 3

The third and final experiment evaluates the generalizability of models trained on the Sen12Landslides dataset across diverse geographic regions. For this purpose, the landslide inventories listed in Table 1 were grouped into six clusters based on geographic proximity (Table 6). The America cluster includes inventories from Alaska, Puerto Rico, and Dominica. The Europe cluster contains only the Italian inventory. Africa is represented by the Chimanimani region in Zimbabwe. The Central Asia cluster includes data from China, the Japanese regions of Hokkaido and Hiroshima, and two areas in Kyrgyzstan. The Southeast Asia cluster comprises inventories from Itogon and Lanao Del Norte in the Philippines, Indonesia, Thrissur in India, and Nepal. Finally, the Oceania cluster consists of data from New Zealand.

**Table 6:** Geographically clustered leave-one-out validation results using Sentinel-2 data and 3D-UNet, including train/test split sizes. “Full” refers to the original unbalanced test sets, while "filtered" refers to test sets with 80% of non-annotated patches removed to improve class balance.

Cluster	Train / Test (full / filtered)	Precision	Recall	F1-Score
America	12242 / 1386 / 681	0.62 ± 0.01	0.53 ± 0.00	0.54 ± 0.01
Europe	8307 / 5321 / 3325	0.58 ± 0.05	0.54 ± 0.03	0.55 ± 0.04
Africa	12495 / 1133 / 558	0.68 ± 0.00	0.71 ± 0.03	0.70 ± 0.01
Central Asia	10333 / 3295 / 1992	0.80 ± 0.03	0.54 ± 0.01	0.56 ± 0.01
Southeast Asia	12308 / 1320 / 782	0.85 ± 0.02	0.57 ± 0.01	0.61 ± 0.02
Oceania	12455 / 1173 / 692	0.74 ± 0.05	0.52 ± 0.01	0.52 ± 0.02

It must be acknowledged that such clustering does not inherently imply the presence of analogous environmental or climatic conditions. A substantial proportion of the inventories contained within a given cluster are still distributed across a wide range of terrains and ecosystems. In certain scenarios, a more sophisticated grouping strategy, such as one based on environmental similarity or landscape attributes, might have yielded more meaningful results. However, for the sake of simplicity and reproducibility, a rudimentary geographic division was employed. In a leave-one-cluster-out configuration, the training process was executed by sequentially excluding one geographic cluster at a time, with subsequent evaluation of the model’s performance on the omitted cluster. This approach emulates a real-world scenario in which local training data for a region is not available.

To minimize training time while maintaining a strong benchmark, this experiment was limited to the 3D-UNet model on Sentinel-2 data. In prior experiments, 3D-UNet exhibited consistent and reliable performance on Sentinel-2 imagery, while demonstrating greater training efficiency compared to more complex architectures such as U-ConvLSTM. During the preliminary testing phase, it was observed that the ratio of annotated to non-annotated samples in the test sets hindered the interpretability of the results. A data filtration process was implemented to address this challenge, removing 80% of the non-annotated patches in the test set. This approach ensured that the metrics more accurately reflected the model’s performance on the relevant annotated areas.

As demonstrated in Table 6, the model is able to detect landslides in unexplored regions, with F1 scores ranging from 0.52 in Oceania to 0.70 in Africa. The higher standard deviations, especially in Europe and Oceania, likely stem from the presence of only a single inventory in each region, limiting environmental diversity and making the models more sensitive to minor training variations. Additionally, it is conceivable that variations in cluster size, annotation density, and landscape heterogeneity may also contribute to the observed variability in the F1 scores of the clusters. The results suggest that the generalization capability could be further enhanced by incorporating region-specific processing strategies, such as tailored normalization, data augmentation, or loss weighting. In summary, this experiment highlights the potential of the Sen12Landslides dataset to facilitate the development of landslide detection models that are globally robust, even in the absence of region-specific training data.

**Fig. 6:**
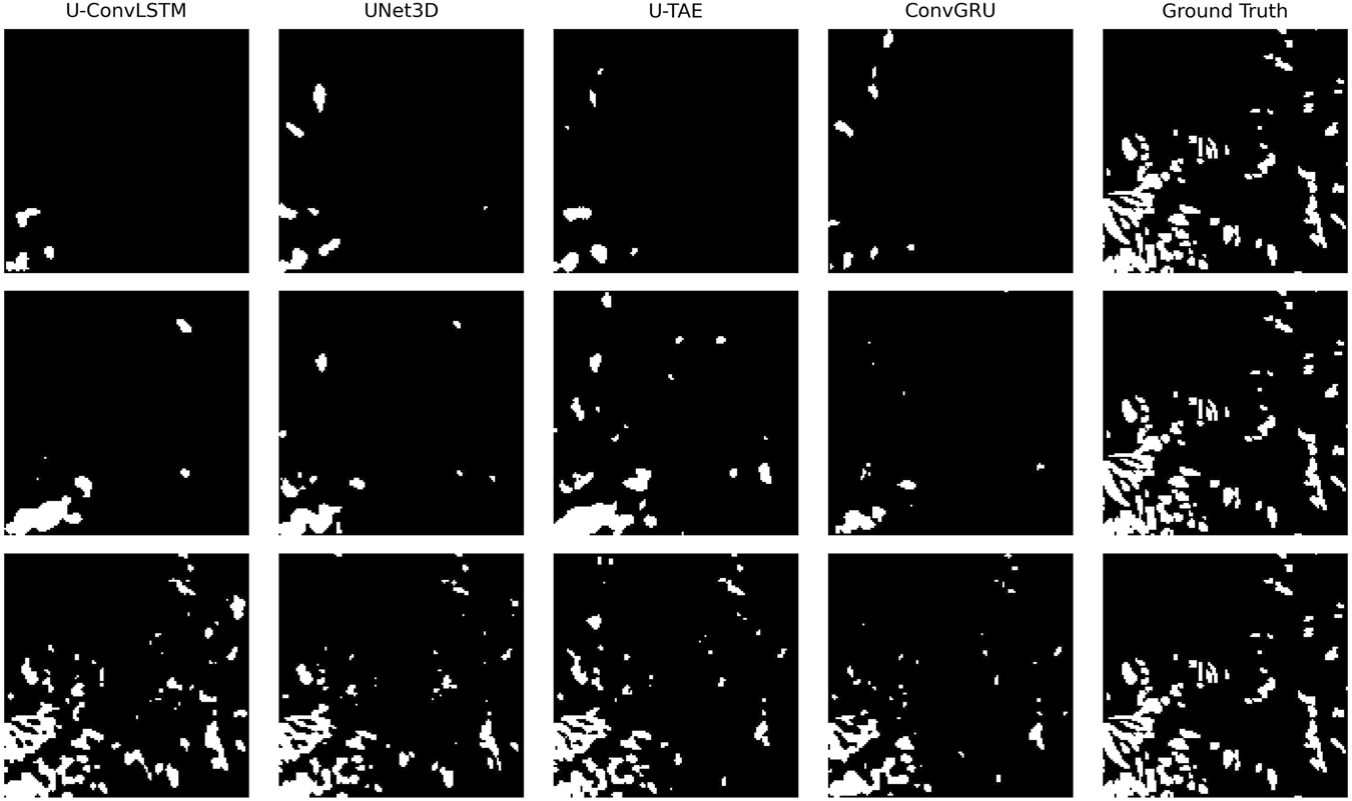
Visual comparison of model predictions against ground truth for Sentinel-1 ascending (top row), Sentinel-1 descending (middle row), and Sentinel-2 (bottom row) inputs from the Sen12Landslides dataset.

To complement the quantitative evaluation, we analyzed visual predictions from each modality and model, as shown in Fig. 6. The figure highlights the qualitative performance of the four deep learning models on the refined Sen12Landslides dataset using Sentinel-1 ascending, Sentinel-1 descending, and Sentinel-2 inputs. The first two rows demonstrate that Sentinel-1 data alone fails to capture many landslides, whereas Sentinel-2 yields significantly better predictions. Among all models, U-ConvLSTM applied to Sentinel-2 comes closest to the ground truth. These visual observations align with the performance metrics and suggest that combining Sentinel-1 and Sentinel-2 inputs could enhance detection by compensating for modality-specific omissions.

#### Summary

This work introduces Sen12Landslides, a large-scale, open-access dataset for landslide detection research and spatio-temporal anomaly analysis. The dataset contains about 75,000 annotated landslide events and over 12,000 standardized image patches from diverse regions worldwide. It integrates time series of Sentinel-1 SAR imagery, Sentinel-2 optical data, the Copernicus DEM, and detailed metadata. This multi-modal, multi-temporal resource enables deep learning models to capture complex spatial patterns and temporal dynamics. Experiments show the benefits of refined labels, temporal depth, and geographic diversity, and demonstrate the dataset’s capacity to support training across multiple models. It can also serve as a benchmark for Earth observation foundation models^52^, environmental analysis, geohazard monitoring, and climate-related research.

## Data Availability

The Sen12Landslides dataset, which is the focus of this study, is publicly available on Hugging Face at: https://huggingface.co/datasets/paulhoehn/Sen12Landslides. The repository includes detailed documentation on its structure and usage, enabling full reproducibility of the experiments described in this paper. In addition to Sen12Landslides, we make use of several previously published datasets. A complete list of these dataset sources, together with accession links and references, is provided in Table 1, ensuring transparency and accessibility of all resources incorporated in this work.

## Code availability

The code used in this study, especially for accessing, processing, and modifying the dataset, will be made publicly available via a dedicated GitHub repository: https://github.com/PaulH97/Sen12Landslides. The repository will include a comprehensive README file to guide users through setup, usage, and potential modifications.
